# The Effect of Microstructure and Axial Tension on Three-Point Bending Fatigue Behavior of TC4 in High Cycle and Very High Cycle Regimes

**DOI:** 10.3390/ma13010068

**Published:** 2019-12-21

**Authors:** Xuechun Bao, Li Cheng, Junliang Ding, Xuan Chen, Kaiju Lu, Wenbin Cui

**Affiliations:** Aeronautics Engineering college, Air Force Engineering University, Xi’an 710038, China; cheng-qiaochu@foxmail.com (L.C.); 18700457532@163.com (J.D.); chenxuan186@gmail.com (X.C.); kaiju.lu@hotmail.com (K.L.); yy2437704026@163.com (W.C.)

**Keywords:** TC4, microstructure, axial tension, three-point bending, very high cycle fatigue

## Abstract

The effects of microstructure and axial tension on the fatigue behavior of TC4 titanium alloy in high cycle (HCF) and very high cycle (VHCF) regimes are discussed in this paper. Ultrasonic three-point bending fatigue tests at 20 kHz were done on a fatigue life range among 10^5^–10^9^ cycles of the alloys with equiaxed, bimodal and Widmanstatten microstructures. Experimental results without axial tension show that three typical shapes of S-N curves clearly present themselves for the three different microstructures. Moreover, the crack initiation sites abruptly shifted from surface to subsurface of the specimen in the very high cycle fatigue regime for equiaxed and bimodal microstructures. But for the Widmanstatten microstructure, both surface and subsurface crack initiation appeared in the high cycle fatigue regime, and the multi-points crack initiation was found in the bimodal microstructure. The subsurface fatigue crack originated from the α_p_ grains in equiaxed and bimodal microstructures. However, it originated from the coarse grain boundary α in the Widmanstatten microstructure. Additionally, the S-N curve shape, fatigue life and fatigue crack initiation mechanism with axial tension are similar to that without axial tension. However, the crack origin point shifts inward with axial tension.

## 1. Introduction

Many studies have shown that there is no endurance limit for most materials in a very high cycle (VHCF) regime [1,2,3,4,5,6]. According to the traditional endurance limit design theory, mechanical design cannot ensure safety. As the requirements for life of aero engines continue to increase, the number of stress cycles for rotating components has already exceeded 10^7^. Bathias pointed out in his monograph that the number of cycles of gas turbine engine components can reach 10^10^–10^11^ cycles [7]. As life expectancy increases, aero engines will face increasingly severe challenges in the field of very high cycle fatigue. It is necessary to study the VHCF performance of aero-engine blade materials.

Titanium alloys are widely used in compressor blades and bladed discs of aerospace engines due to their high specific strength, low density, excellent corrosion resistance and good heat resistance [8,9,10]. With the wide applications of titanium alloys in the aerospace industry, research on VHCF property of titanium alloys is also on the increase. Similar to steel [11,12], aluminum alloy [13,14] and cast iron [15,16], the crack initiation point of titanium alloy also shifts from the surface to internal or subsurface initiation in VHCF regime [6,10]. Liu et al. [17] carried out the VHCF test of Ti–6Al–4V titanium alloy under different stress ratios. The results show that the fatigue S-N curves exhibit different characteristics under different stress ratios, and the fatigue life increases with increasing stress ratio. Meanwhile, it was also found that as the stress drops, the surface slip mechanism of high cycle fatigue changes to internal cleavage mechanism of VHCF, and facets formed by primary α grains cleavage play an important role in fracturing. Li et al. [18] and Yang et al. [19] also reached the same conclusion. Heinz et al. [20] found that the proportion of β phase in an optically dark area (ODA) was very low by the analysis of Ti-6Al-4V fracturing in a VHCF regime. The scanning electron microscope (SEM) analysis of the face passing through the ODA region revealed that microcracks were initiated in the primary α phase, which were about 45° from the loading direction, and the grain refinement was found by electron back scatter diffraction pattern (EBSD) analysis. Pan et al. [10] and Liu et al. [21] investigated the VHCF behavior of Ti-6Al-4V alloy by the means of transmission electron microscope (TEM). At the negative stress ratio, there is a nanocrystalline layer in the rough surface area (RA). Nevertheless, there is no nanocrystal in the non-negative stress ratio. This view is consistent with the numerous cyclic pressing (NCP) model proposed by Hong [22].

Numerous studies have shown that the microstructure characteristics have a significant impact on the fatigue behavior of titanium alloys [23,24,25,26,27]. The investigation by Chandran et al. [28] indicated a significant difference in very high cycle behavior between two Ti-22V-4Al alloys, whose proportions of primary α phase were 10% and 45%, respectively. The observations by Liu et al. [29] revealed that the content of primary α phase (50% and 95%) had no obvious influence on the very high cycle property of Ti-6Al-4V alloy. Zuo et al. [30] analyzed the crack initiation of Ti-6Al-4V alloy with both basketweave and bimodal microstructures and indicated that the internal fatigue crack initiation was caused by microstructural inhomogeneity. In bimodal microstructure, cracks originated from α_p_ grains or the boundaries of α_p_ grain and crack initiation points became the colonial boundaries or α–β interfaces in basketweave microstructure. Crupi et al. [31] obtained similar results through investigating the VHCF behavior of Ti-6Al-4V alloy produced by two forging processes. Recently, Nie et al. [32] studied the fatigue performance of TC21 titanium alloy with two sizes of basketweave in the life span of 10^5^–10^9^. They pointed out that the fatigue property of the short size basketweave is higher. However, the origin mechanism of fatigue cracks in the two materials was semblable.

It is worth noting that the research results above were carried out by uniaxial tension or rotating bending. The VHCF properties under bending vibration loading for the titanium alloys have not been very well understood till now. However, the loading mode of aeroengine blades is bending vibration. aeroengine blades also bear centrifugal tensile stress. Scholars are yet to conduct material fatigue tests with both vibration stress and centrifugal tensile stress. Therefore, it is necessary to carry out relevant research. TC4 is an α + β titanium alloy widely used in the manufacturing of aeroengine compressor blades. In this study, the three-point bending fatigue in HCF and VHCF regimes of a TC4 alloy was investigated. The centrifugal force on aero engine blades was simulated by applying axial tension. First, ultrasonic fatigue tests at 20 kHz and room temperature were performed on the tested TC4 alloy with equiaxed, bimodal and Widmanstatten microstructures. The effect of microstructure on fatigue life was obtained by comparing the fatigue S-N curve. The fatigue crack initiation mechanism is revealed using SEM and energy dispersion spectrum (EDS) analyses. Besides, the reason why the crack origin point shifts inward with axial tension is revealed herein.

## 2. Materials and Methods

### 2.1. Materials

**Figure 1:**
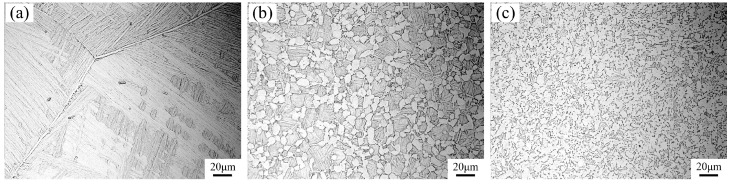
Microstructures of TC4 alloy: (**a**) Widmanstatten; (**b**) bimodal; and (**c**) equiaxed.

**Figure 2:**
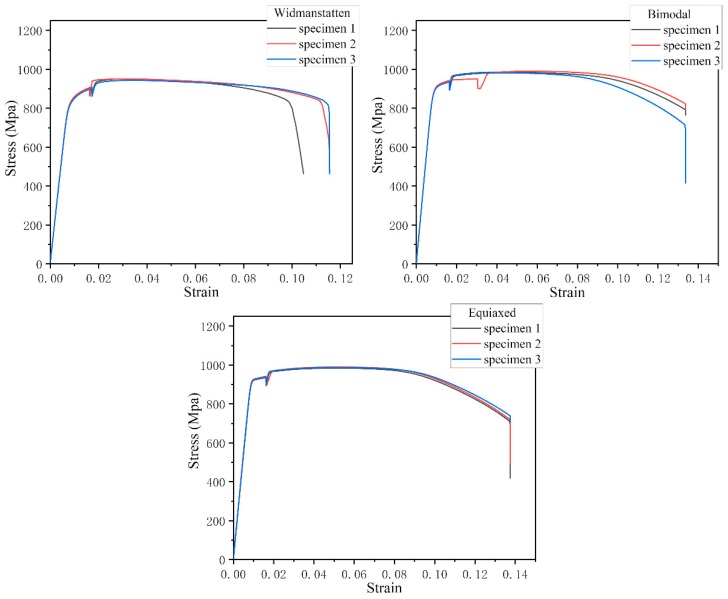
Tensile stress–strain curve of TC4 titanium alloy with three different microstructures.

The fatigue tests were carried out on a TC4 titanium alloy with the chemical composition of Ti-6Al-4V. Three microstructures of Widmanstatten, bimodal and equiaxed alloy were obtained by different thermal treatment procedures. The procedures for heat treatment were: (1) 1030 °C for 1 h + air quenching, and then 530 °C for 4 h + air quenching; (2) 920 °C for 1 h + air quenching, and then 530 °C for 4 h + air quenching; and (3) 700 °C for 2 h + air quenching. Figure 1 shows the three microstructures of the TC4 used in this study. The crystal grains in the metallographic pictures were visible and clear. No segregation, folding, cracks, inclusions or serious defect areas were observed. The Widmanstatten microstructure consisted of lamellar tissue. The primary α phase volume fraction of the bimodal microstructure was about 40% and 90% in the equiaxed microstructure. The tensile mechanical properties at room temperature were tested at a strain rate of 0.00025/s (measurement data is listed in Table 1, and the tensile stress–strain curve is represented in Figure 2).

### 2.2. Experimental Methods

**Figure 3:**
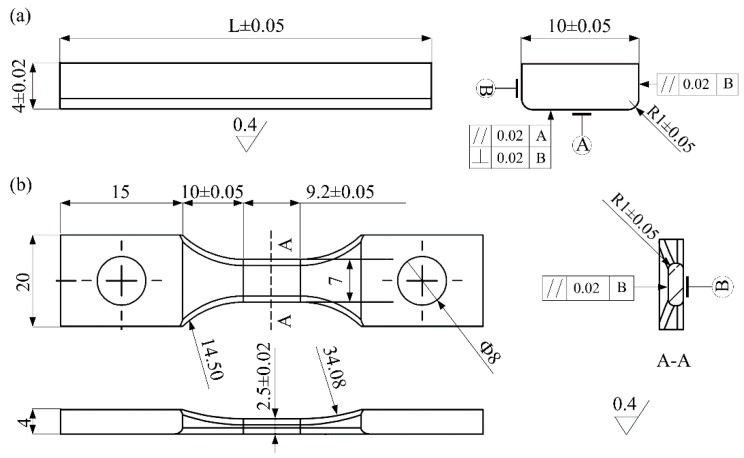
Shape and dimensions (mm) of three-point bending specimens. (**a**) without axial tension; (**b**) with axial tension.

**Figure 4:**
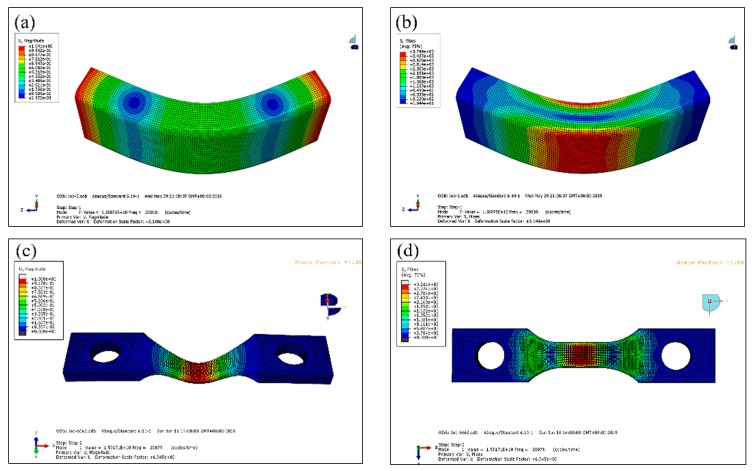
Displacement–stress field of the specimens. (**a**,**b**) Widmanstatten microstructure without axial tension; (**c**,**d**) bimodal microstructure with axial tension.

**Figure 5:**
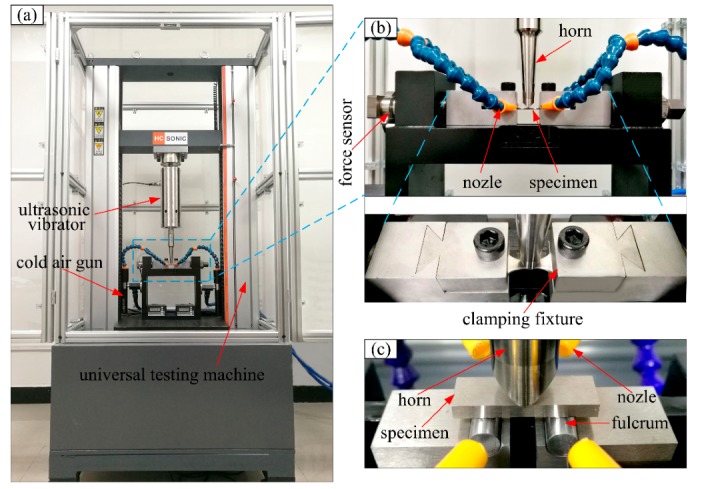
Test equipment and loading methods. (**a**) Overall picture of the testing machine; (**b**) three-point bending load with axial tension; (**c**) three-point bending load without axial tension.

The fatigue experiment was carried out using an ultrasonic three-point bending fatigue test system (HC SONIC, Hangzhou, China) with a load frequency of 20 kHz. The ultrasonic fatigue test is based on resonance principle: the resonance frequency of three-point bending ultrasonic fatigue sample is identical with that of the test facility. The shape and dimensions (mm) of three-point bending specimen in this study are shown in Figure 3, and the values of L shown in Figure 3a change with material properties. In this test, in order to obtain the same resonant frequency, the L values of Widmanstatten, bimodal and equiaxed microstructures, were 31.4, 31.2 and 31 mm, respectively. The specimen capable of applying axial tension with bimodal microstructure is shown in Figure 3b. Moreover, the chamfer of R = 1 mm was processed at the bottom corner of the specimen, which eliminates the stress concentration, and greatly improves the accuracy of the test results. In order to acquire the natural frequency and displacement–stress fields in the specimen, the modal analysis of the three-point specimen was conducted using finite element software; for example, the displacement-stress field of the specimen without axial tension and with the Widmanstatten microstructure are shown in Figure 4a,b. The resonant frequency of the specimen was 20,010 Hz, which is very close to 20 kHz. From the displacement field diagram for the side of the specimen in Figure 4a, two symmetrically distributed displacement stagnation points can be clearly seen. When the three-point bending ultrasonic fatigue test is carried out, the two fulcrum positions are present (displacement stagnation points). According to the different colors of the specimen stress field, it was found that there was a maximum tensile stress area at the bottom section, and the maximum compressive stress existed on the upper surface, as shown in Figure 4b. The ratio of the vibration bending normal stress and the vibration displacement at the bottom of the specimen is equal to the numerical value of stress-displacement coefficient. The displacement-stress field of the specimen capable of applying axial tension with bimodal microstructure is shown in Figure 4c,d. Both ends of the specimen were clamped and the axial tensile stress was 200 MPa. The resonant frequency of the specimen was 20,079 Hz and there is a maximum tensile stress area at the bottom section, represented with a red color in Figure 4d. Pictures of the test machine and loading method are shown in Figure 5. The control error of universal testing machine was 0.5%, and the output error of vibration displacement was less than 1 μm. While conducting the three-point bending test with axial tension, a specific clamp was used to fix the specimen and to apply axial tensile force, as shown in Figure 5b. The bolt on the right was used to apply tensile force, and the force sensor on the left was used to monitor the tensile force.

All fatigue tests were performed in air, at room temperature, and with a stress ratio of *R* = 0.4. In order to eliminate the rise in temperature of specimens due to ultrasonic vibration, compressed cold air was used for cooling, and the maximum temperature of the test section was kept below 40 °C. The value of axial tension in this test was 200 MPa. One or two specimens were tested per load level and the number of samples increased to where the trend of the S-N curve was not obvious. The spans between two fulcrums for Widmanstatten, bimodal and equiaxed microstructures are 17.1 mm, 17.0 mm and 16.9 mm, respectively. The MTI-2100 optical fiber displacement sensor was used for calibrating the ultrasonic vibration fatigue system and to measure the maximum displacement at the bottom of specimen, where the measurement range is in 1–199.9 μm. The measure precision reached 0.1 μm, and the measured frequency range around 1–150 kHz. Meanwhile, the fracture surfaces of the failed specimens were examined using a JSM-6460 scanning electron microscope (JEOL, Tokyo, Japan).

## 3. Results

### 3.1. S-N Characteristics

**Figure 6:**
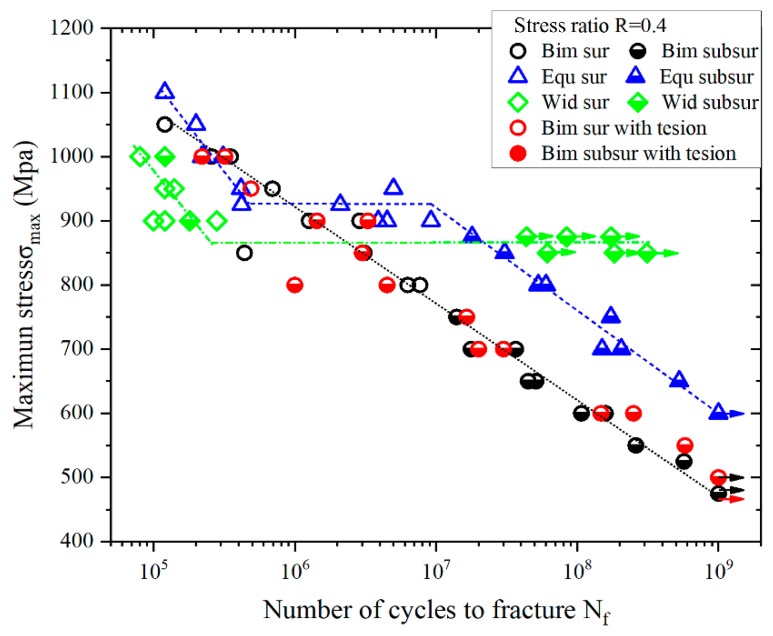
S-N curves of three TC4 titanium alloy materials with different microstructures (arrows denote the run-out specimens).

The S-N curve in high cycle and very high cycle fatigue regimes over the range of 10^5^–10^9^ cycles for the three TC4 titanium alloy materials was obtained (Figure 6). The “maximum stress” in a S-N curve stands for the maximum tensile stress at the bottom of the specimen. The calculation formula for maximum stress is σ_max_ = σ*_m_* + σ*_u_*, where σ*_m_* stands for the mean stress, and σ*_u_* stands for the vibration stress amplitude. And the possible yielding and stress redistribution was not considered in this case. The diamond, circular and triangular dots represent the fatigue lives of Widmanstatten, bimodal and equiaxed microstructures, respectively. The solid symbols in the figure indicate that the fatigue crack is initiated on the surface of the specimen, and the semi-solid stands for subsurface initiation. For both bimodal and equiaxed microstructures without axial tension, the fatigue crack was initiated on the surface of the specimen in HCF regime, and the crack origin point abruptly shifted from the surface to subsurface in VHCF. However, surface and subsurface crack initiations coexisted in the HCF range for the Widmanstatten microstructure and bimodal microstructure with axial tension.

**Figure 7:**
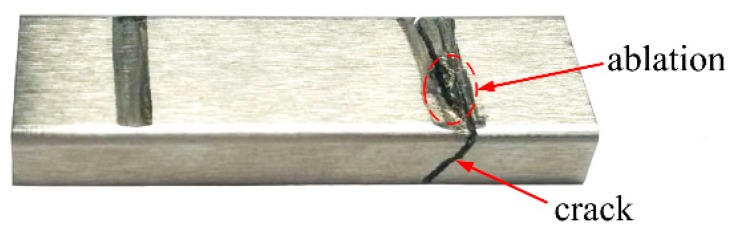
Photo of specimen broken from the fulcrum position of Widmanstatten microstructure.

It is obvious that three completely different shapes can be observed from the S-N curve. The bimodal structure is a continuous declining shape. With the decrease of the maximum stress, the S-N curve shows a linear downward trend. For the equiaxed microstructure, the S-N curve presents as a stepwise type. It can be divided into three parts between the fatigue life of 1 × 10^5^ and 1 × 10^9^ cycles. In the first part, with the decreased maximum stress level, the fatigue life increased up to about 5 × 10^5^ cycles; in the second part, the horizontal stress plateau of 925 MPa exists among 5 × 10^5^ to 1 × 10^9^ cycles; third, the S-N curve declined again till 1 × 10^9^ cycles. For the Widmanstatten microstructure, an asymptotic S-N curve appeared. When the maximum stress level was around 860 MPa, it turned horizontally, which is usually called the fatigue limit. Specimens will not fail in case of the maximum stress level being less than the fatigue limit. Regrettably, once its cyclic number reaches 1 × 10^8^ cycles, specimens broke from the fulcrum position, and the test could not be continued. Besides, due to the crack at the fulcrum, the friction at the moment of fracture was intensified, and there was sign of ablation at the fracture location as shown in Figure 7. This phenomenon indicates that the Widmanstatten microstructure is poor at fretting fatigue resistance.

The high cycle bending fatigue performance of the bimodal and equiaxed microstructures is better than that of the Widmanstatten microstructure. In contrast, the very high cycle fatigue property of the Widmanstatten microstructure is the best. When the fatigue life exceeds 10^7^ cycles, a 20% higher fatigue strength is detected from the bimodal to equiaxed microstructure.

### 3.2. Fracture Surface Morphology

In this study, the surface and subsurface crack initiations were discovered in the fracture surfaces of all broken TC4 titanium alloy specimens of the three different microstructures. Moreover, some specimens were observed with multiple fatigue crack initiation regions or multiple crack origin points in a single crack initiation region.

**Figure 8:**
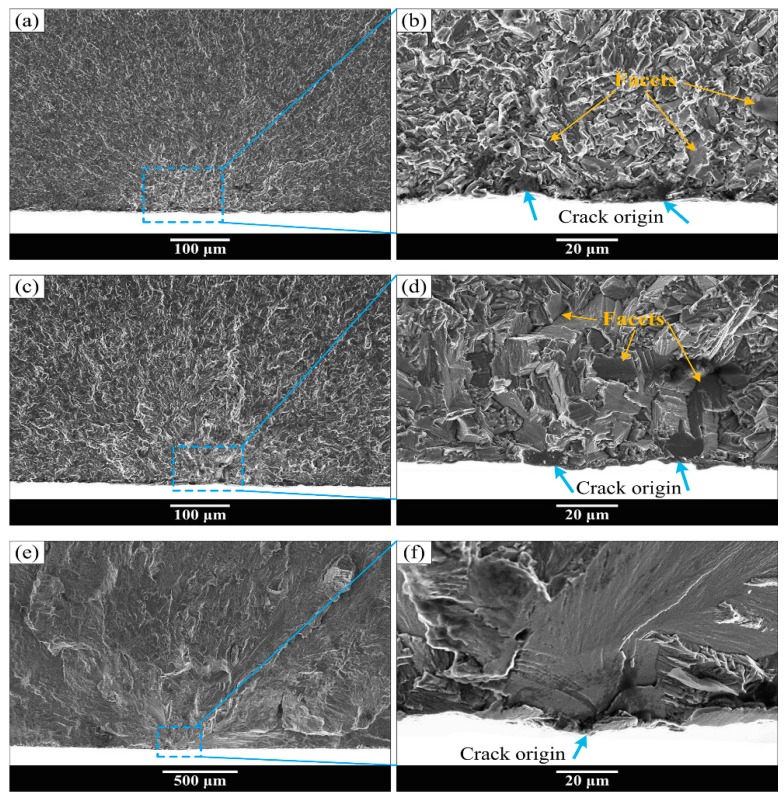
Typical fatigue fracture surface SEM morphology of “surface crack initiation.” (**b,d**,**f**) The enlarged images at crack initiation regions of (**a**,**c**,**e**), respectively. (**a**,**b**) equiaxed microstructure, σ_max_ = 900 MPa, N_f_ = 9.21 × 10 ^6^ cycles; (**c**,**d**) bimodal microstructure, σ_max_ = 1000 MPa, N_f_ = 2.58 × 10^5^ cycles; (**e**) and (**f**) Widmanstatten microstructure, σ_max_ = 950 MPa, N_f_ = 1.19 × 10^5^ cycles.

**Figure 9:**
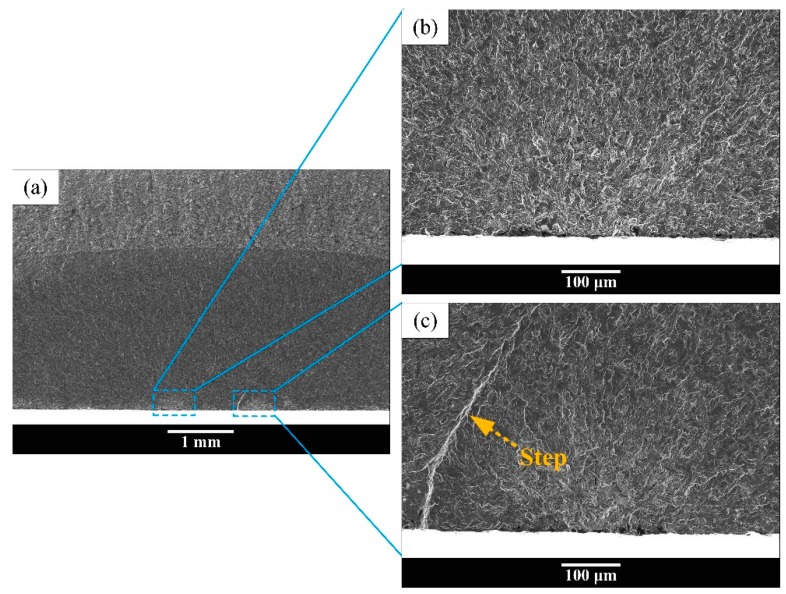
Multiple crack initiation regions in bimodal microstructure at σ_max_ = 900 MPa, N_f_ = 4.38 × 10^5^ cycles. (**a**): Low magnification morphology; (**b**,**c**): high magnification images of crack initiation regions.

Typical fatigue fracture surface SEM morphology of “surface crack initiation” is exhibited in Figure 8 and Figure 9. All of the fractures of equiaxed microstructure and most of the fracture surface of bimodal and Widmanstatten microstructures were detected to have surface crack initiation with single crack initiation region in high cycle fatigue regime as shown in Figure 8. Figure 8b,d,f shows the high magnification images at crack initiation regions of Figure 8a,c,e, respectively. The light-colored river-like crack propagation paths are presented in Figure 8a,c,e, and the crack origin point was determined based on the convergence point of fatigue crack propagation paths. Multiple crack origin points and facets were found in equiaxed and bimodal microstructures, as indicated by the arrows in Figure 8b,d. It is obvious that the cracks originated from surface slip or surface grain cleavage. However, the Widmanstatten microstructure only shows a single crack origin point (Figure 8f). Meanwhile, the failure mode of “multiple fatigue crack initiation regions” was observed in the specimens of bimodal microstructure, as shown in Figure 9. The high magnification images of the two crack initiation regions in Figure 9a are shown in Figure 9b,c. The step morphology is marked by an orange arrow in Figure 9c. The formation of step morphology on this fracture surface was caused by the meeting of cracks from the two crack initiation regions on either side of the step. Due to multiple fatigue crack initiation regions, the fatigue life of the specimen is significantly shorter.

**Figure 10:**
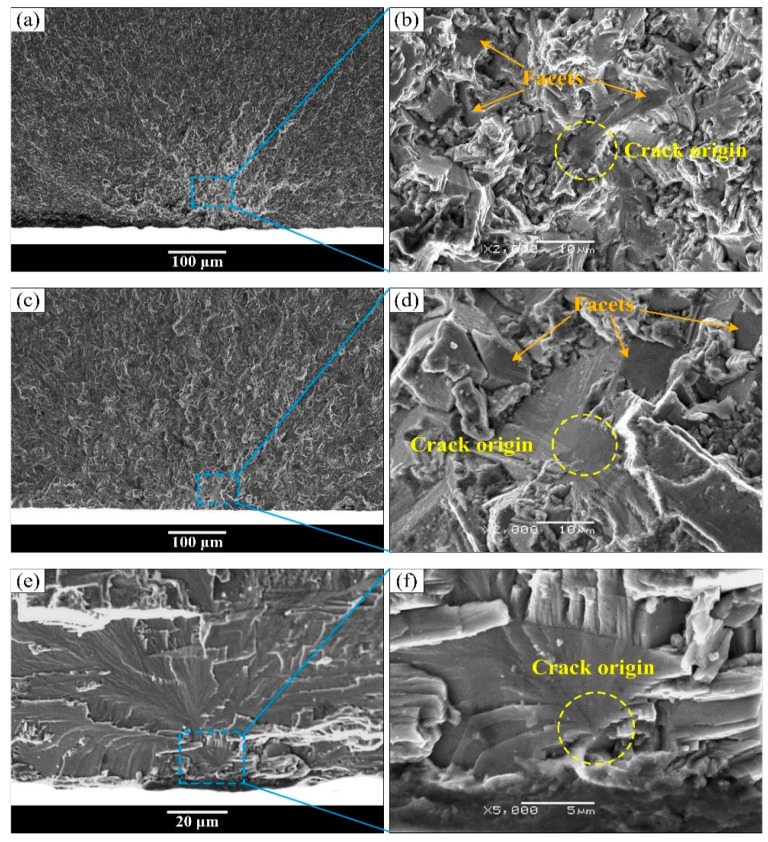
Typical fatigue fracture surface SEM morphology of “subsurface crack initiation.” (**b**,**d**,**f**) The enlarged images at crack initiation regions of (**a**,**c**,**e**), respectively. (**a**,**b**) equiaxed microstructure, σ_max_ = 750 MPa, N_f_ = 1.73 × 10^8^ cycles; (**c**,**d**) bimodal microstructure, σ_max_ = 600 MPa, N_f_ = 1.68 × 10^8^ cycles; (**e**,**f**) Widmanstatten microstructure, σ_max_ = 900 MPa, N_f_ = 1.99 × 10^5^ cycles.

**Figure 11:**
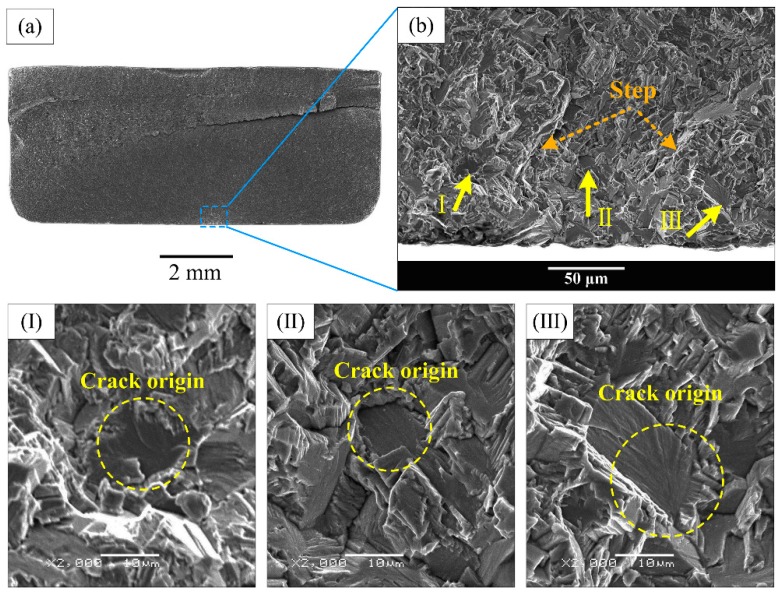
Multiple crack origin points in bimodal microstructure at σ_max_ = 600 MPa, N_f_ = 1.08 × 10^8^ cycles. (**a**): Low magnification morphology (**b**): high magnification image of crack initiation region.

However, all the fracture surfaces of equiaxed and bimodal microstructures were detected to have subsurface crack initiation in the very high cycle fatigue regime, and the subsurface crack initiation morphology also exists in the high cycle fatigue fracture surface of the Widmanstatten microstructure. Typical fatigue fracture surface SEM morphology of “subsurface crack initiation” is exhibited in Figure 10 and Figure 11. Figure 10a,c,e shows the overall morphology of crack initiation regions, and high magnification images at crack initiation regions are shown in Figure 10b,d,f, respectively. It can be seen in Figure 10a,c,e that the origination of river-like crack propagation paths is a point on the subsurface of the specimen. Therefore, this failure mode is known as “subsurface crack initiation.” The facets that were pointed out by the orange arrows appeared in the crack initiation region of equiaxed and bimodal microstructures, as shown in Figure 10b,d. Meanwhile, the crack origin point was pointed out by the yellow circle. It can be clearly seen that the crack origin point is mainly a facet in the fracture surface of equiaxed and bimodal microstructures and a quasi-smooth facet for the Widmanstatten microstructure. Multiple crack origin points in single crack initiation region of bimodal microstructure were also observed (Figure 11). The step morphology caused by the meeting of fatigue cracks is marked in Figure 11b. The crack initiation points are quasi-smooth facets circled by a yellow dotted line, as shown in Figure 11I–III.

## 4. Discussion

### 4.1. Fatigue Crack Initiation Mechanism

**Figure 12:**
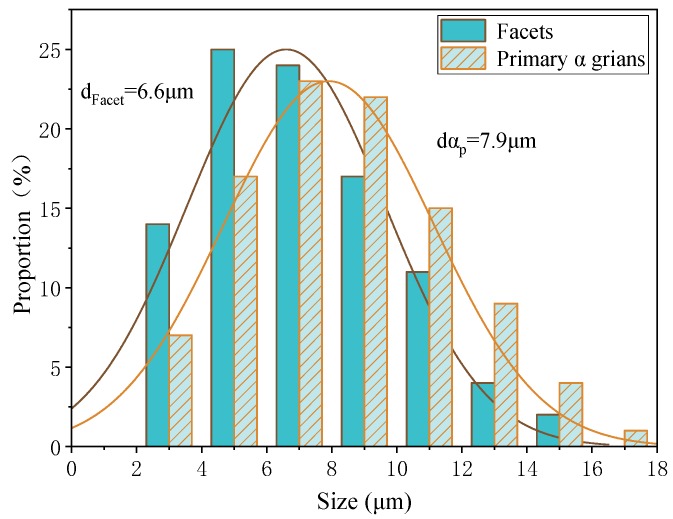
The size distributions of α_p_ grains and facets of equiaxed microstructure.

**Figure 13:**
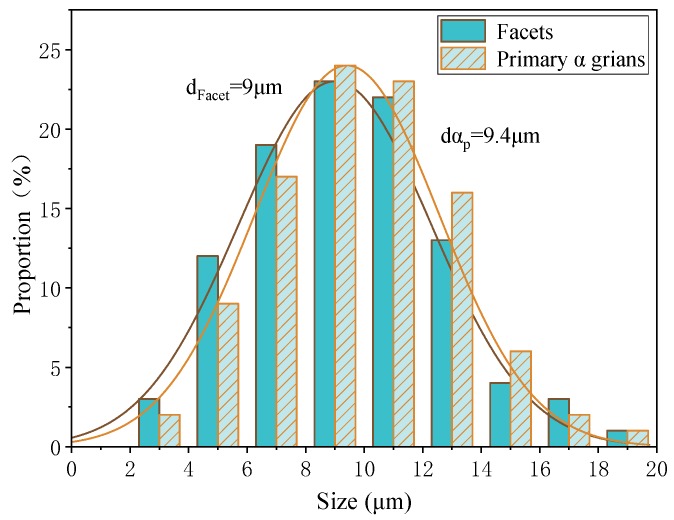
The size distributions of α_p_ grains and facets of bimodal microstructure.

For the equiaxed and bimodal microstructures, abundant facets were discovered in the fatigue crack initiation regions. Some surface and all subsurface origin points were facets. In order to explore the fatigue crack initiation mechanism, it was necessary to perform research on the characteristics of facets. According to the existing results of uniaxial tension or rotating bending, the cracking of α_p_ grains is the cause of facets in titanium alloys [18,19]. For the purpose of revealing the formation mechanism of a facet in this test, the size of the facets and the α_p_ grains were counted, and the results are plotted in Figure 12 and Figure 13. The dimensional data shown in the figure is the length of the longest axis. The α_p_ grains were measured from metallographic and fracture surface SEM morphology. On the one hand, both statistics have similar distribution. On the other hand, the average size of the facets is close to the α_p_ grains, and the size of the facet is slightly smaller than the size of an α_p_ grain. This statistical regularity is consistent with the results of the very high cycle fatigue test of titanium alloy under other loading modes [17,19,21]. Therefore, it can be speculated that the facets are products from the cleavage of the α_p_ grains. Besides, the average size of α_p_ grain of equiaxed microstructure (7.9 μm) is smaller than that of bimodal (9.4 μm), so the fatigue life of equiaxed microstructure is longer than that of bimodal microstructure in the VHCF regime shown in Figure 6.

**Figure 14:**
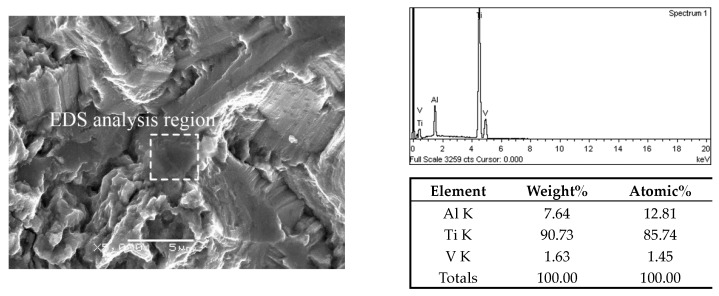
EDS analysis result on crack origin point of equiaxed microstructure, σ_max_ = 750 MPa, N_f_ = 1.73 × 10^8^ cycles.

**Figure 15:**
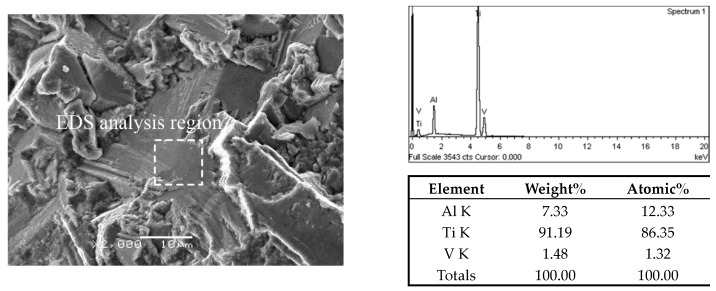
EDS analysis result on crack origin point of bimodal microstructure, σ_max_ = 600 MPa, N_f_ = 1.68 × 10^8^ cycles.

**Figure 16:**
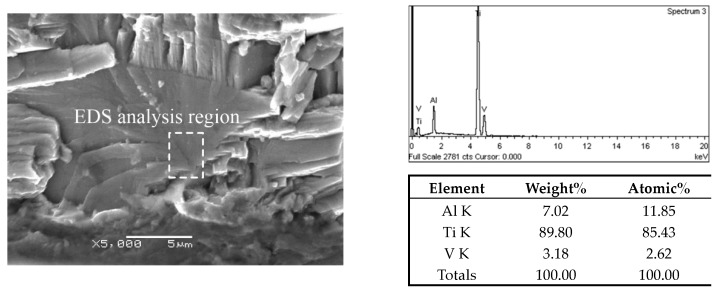
EDS analysis result on crack origin point of the Widmanstatten microstructure, σ_max_ = 900 MPa, N_f_ = 1.99 × 10^5^ cycles.

In addition, EDS analysis was performed on the facets of the crack origin points to confirm the initiation mechanism of fatigue crack. For TC4 titanium alloy, elemental Al was the α-phase stabilizer, and elemental V was the β-phase stabilizer [33,34]. The EDS results on crack origin point of the three microstructures are shown in Figure 14, Figure 15 and Figure 16. It can be distinctly seen from the analysis results that the aluminum content of the facet and quasi-smooth facets at the origin point of the crack was higher than the average value of the material, and the content of V element was below average. Therefore, the facets in the fracture surface of equiaxed and bimodal microstructures formed due to the cleavage of α grains, and the quasi-smooth facets of the crack origin points of the Widmanstatten microstructure formed due to the cracking of the α colony.

From the above discussion, it can be concluded that the fatigue cracks in subsurface, and some surface initiations with facets of equiaxed and bimodal microstructures, originated from the cleavage of α_p_ grains. Meanwhile, some studies suggest that the crack origin region of titanium alloy possess specific spatial and crystallographic orientations [19,25]. For the Widmanstatten microstructure, it was easy to cause stress concentration at the coarse grain boundary α, as shown in Figure 1a. Therefore, it can be inferred that the fatigue cracks in subsurface initiation of Widmanstatten microstructure originated from the quasi-cleavage fracture of the coarse grain boundary α.

### 4.2. The Influence of Axial Tensile Stress

**Figure 17:**
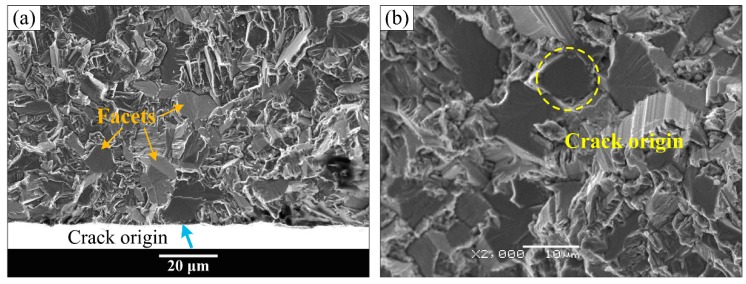
Typical fatigue fracture surface SEM morphology of bimodal microstructure with axial tension. (**a**) “Surface crack initiation” at σ_max_ = 800 MPa, N_f_ = 4.5 × 10^6^ cycles; (**b**) “subsurface crack initiation” at σ_max_ = 600 MPa, N_f_ = 1.47 × 10^8^ cycles.

Firstly, the application of axial tensile stress has no effect on the shape of fatigue S-N curve and fatigue life, as shown in Figure 6. Secondly, the mechanism of fatigue crack initiation is similar under the two stress conditions. The typical fatigue fracture surface SEM morphologies of “surface crack initiation” and “subsurface crack initiation” of bimodal microstructures with axial tensile are shown in Figure 17. It is obvious that the fatigue crack initiated from the surface and subsurface both were caused by the cleavage of α_p_ grains. 

**Figure 18:**
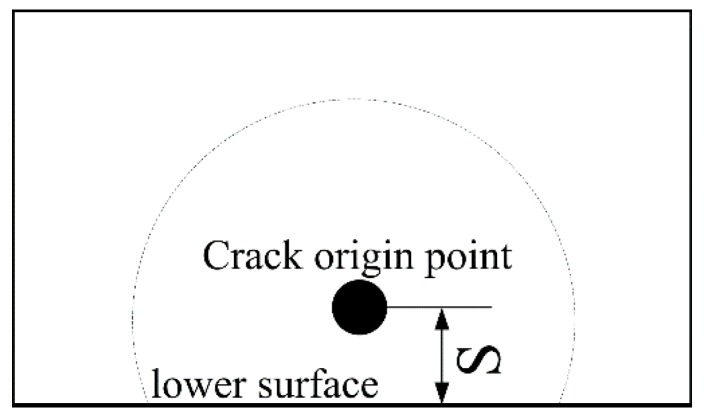
The schematic of S (the distance between the origin point and the lower surface of the sample).

**Figure 19:**
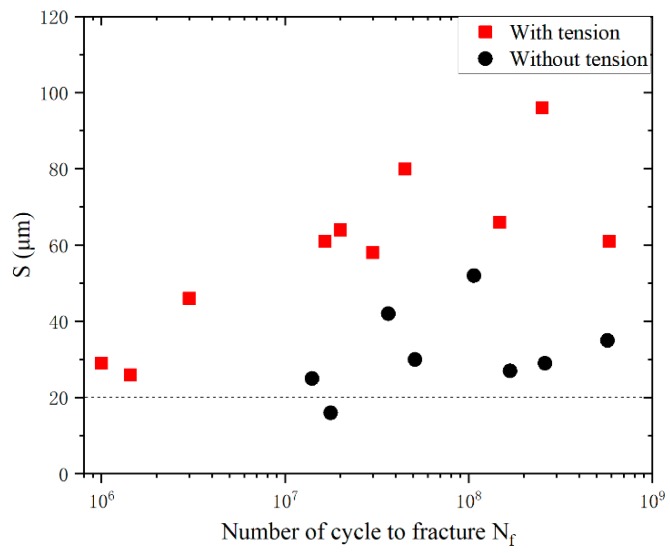
The statistical results of S (the distance between the origin point and the lower surface of the sample).

Thirdly, subsurface crack initiation occurs in the high cycle fatigue regime with the application of axial tensile stress, and the crack origin point seems to shift inwards. Thus, the statistics of the distance (S) between the origin point and the lower surface of the specimen were carried out. The schematic of S is shown in Figure 18 and the statistical results are shown in Figure 19. With the increase of fatigue life, the value of S increases. The value of S with axial tensile stress is clearly greater than that without axial tensile stress, which indicates that the crack origin point did shift inward. Moreover, the minimum value of S is about 20 μm, which is about the size of two α_p_ grains. Therefore, it can be concluded that with reducing maximum stress, the microcrack in the surface α_p_ grains cannot extend through the second grain, and the fatigue crack cannot initiate on the surface. It can then be transferred to subsurface initiation starting from the third grain. With the increasing stress cycle, the damage accumulates on the subsurface and the fatigue crack is transferred to initiate from the subsurface.

**Figure 20:**
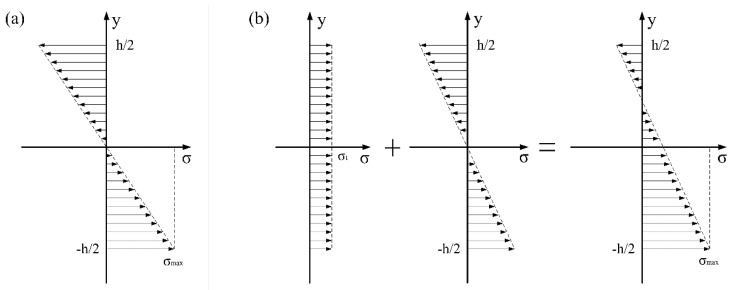
The normal stress in the middle section of the specimen. (**a**) Without axial tension; (**b**) with axial tension.

In order to investigate the reason why behind the crack origin point shifting inward, we analyzed the normal stress in the middle section of the specimen that was analyzed, as shown in Figure 20. Figure 20a shows the stress state of three-point bending without axial tension, while Figure 20b shows the stress state with axial tension. With the application of axial tensile stress, the gradient of normal stress in the middle section decreases. Therefore, when the maximum stress on the surface is the same, the stress inside the specimen increases, which leads to a higher probability of crack initiation in the area further away from the lower surface.

In order to investigate the reason why behind the crack origin point shifting inward, we analyzed the normal stress in the middle section of the specimen, as shown in Figure 20. Figure 20a shows the stress state of three-point bending without axial tension, while Figure 20b shows the stress state with axial tension. With the application of axial tensile stress, the gradient of normal stress in the middle section decreases. Therefore, when the maximum stress on the surface is the same, the stress inside the specimen increases, which leads to a higher probability of crack initiation in the area further away from the lower surface.

## 5. Conclusions

In this paper, the effect of microstructure on three-point bending fatigue behavior of TC4 in high cycle and very high cycle regime was studied. The conclusions were drawn as follows.

1. Three completely different shapes of S-N curve were presented for equiaxed, bimodal and Widmanstatten microstructures among the fatigue lives of 10^5^–10^9^ cycles. They were stepwise, continuously declining and asymptotic types.
2. The bending fatigue performance of the equiaxed and bimodal microstructures was obviously better than that of the Widmanstatten microstructure in the high cycle regime. The very high cycle bending fatigue property of the Widmanstatten microstructure was the best, whereas the fretting fatigue resistance of the Widmanstatten microstructure was poor.
3. The cracks in subsurface and surface initiation with facets of equiaxed and bimodal microstructure originated from α_p_ grains. The cracks in the subsurface of Widmanstatten microstructure originated from the coarse grain boundary α.
4. At the same stress level, the smaller the size of α_p_ grains, the longer the fatigue life of TC4 in the very high cycle regime. Therefore, very high cycle fatigue performance can be improved with the refinement of α_p_ grains.
5. The S-N curve shape, fatigue life and fatigue crack initiation mechanism with axial tension are similar to those without axial tension. But, the crack origin point shifts inward with axial tension.

## Figures and Tables

**Table 1:** Mechanical properties of TC4 alloy for the three microstructures.

Microstructure	Tensile Strength (MPa)	Yield Strength R_p0.2_ (MPa)	Young’s Modulus (GPa)	Elongation (%)
Widmanstatten	949	848	108	10.9
Bimodal	986	909	111	13.1
Equiaxed	987	926	114	13.6
